# Preference for novel biomedical HIV pre-exposure prophylaxis methods among adolescent girls and young women in Kampala, Uganda: a mixed methods study

**DOI:** 10.3389/fpubh.2024.1369256

**Published:** 2024-05-23

**Authors:** Yunia Mayanja, Ivy Kayesu, Onesmus Kamacooko, Jane Frances Lunkuse, Vincent Muturi-Kioi, Matt Price, Kyriaki Kosidou, Anna Mia Ekström

**Affiliations:** ^1^Medical Research Council/Uganda Virus Research Institute and London School of Hygiene and Tropical Medicine (MRC/UVRI & LSHTM) Uganda Research Unit, Entebbe, Uganda; ^2^Department of Global Public Health, Karolinska Institutet, Stockholm, Sweden; ^3^Child Health and Development Centre, School of Medicine, Makerere University, Kampala, Uganda; ^4^IAVI, Nairobi, Kenya; ^5^4IAVI, New York, NY, United States; ^6^Department of Epidemiology and Biostatistics, University of California, San Francisco, San Francisco, CA, United States; ^7^Centre for Epidemiology and Community Medicine, Region Stockholm, Stockholm, Sweden; ^8^Department of Infectious Diseases/Venhälsan, Södersjukhuset, Stockholm, Sweden; ^9^Department of Clinical Science and Education, Södersjukhuset, Stockholm, Sweden

**Keywords:** pre-exposure prophylaxis, biomedical HIV prevention, preference, adolescent girls and young women, Uganda, Eastern and Southern Africa

## Abstract

**Background:**

Novel HIV pre-exposure prophylaxis (PrEP) methods including a potential future HIV vaccine, will increase prevention options for adolescent girls and young women (AGYW) at high risk of HIV infection in Eastern and Southern Africa, yet data on AGYW’s preferences for various PrEP methods is limited. We investigated preferences for five biomedical PrEP methods *(oral, injectable, vaginal ring, implant, HIV vaccine)* among 14–24-years-old AGYW in Kampala, Uganda.

**Methods:**

From January to December 2019, we conducted a mixed methods study including 265 high-risk AGYW. After receiving two education sessions on the five PrEP methods, participants were asked about their “most preferred PrEP method.” Multinomial logistic regression (oral PrEP as reference category) was used to determine participant characteristics associated with method preference. Results are presented as adjusted relative risk ratios (aRRR) with 95% confidence intervals (CI). In-depth interviews were conducted with 20 selected participants to examine reasons influencing PrEP preferences and suggestions for method improvements. Transcripts were analyzed thematically.

**Results:**

Participants preferred methods were: HIV vaccine (34.7%), oral PrEP (25.7%), injectable PrEP (24.9%), PrEP implant (13.6%), and vaginal ring (1.1%). Preference for injectable PrEP increased with every year of age (aRRR 1.22; 95% CI 1.04–1.44) and among participants with chlamydia or gonorrhoea (aRRR 2.53; 95% CI 1.08–5.90), while it was lower among participants having sexual partner(s) living with HIV or of unknown HIV status (aRRR 0.30; 95% CI 0.10–0.91). Preference for PrEP implants also increased with age (aRRR 1.42; 95% CI 1.14–1.77) and was strong among participants having ≥10 sexual partners in the past 3 months (aRRR 3.14; 95% CI 1.16–8.55), while it was lower among those with sexual partner(s) living with HIV or of unknown HIV status (aRRR 0.25; 95% CI 0.07–0.92). PrEP method preference was influenced by product attributes and prior experiences with similar product forms commonly used in health care.

**Conclusion:**

AGYW have varied preferences for biomedical PrEP method and those with higher sexual behavioral risk prefer long-acting methods. As we anticipate more available PrEP options, oral PrEP use should be supported among AGYW, especially for those with sexual partners living with HIV or of unknown HIV status.

## Introduction

Eastern and Southern Africa is the region with the highest HIV prevalence in the world, about 54% of all people living with HIV (1). Adolescent girls and young women (AGYW) below 25 years in this region are at high risk for HIV acquisition, with a mean HIV prevalence estimated to be three times higher than that of their male peers (25% versus 8%, respectively), and, in year 2021, accounted for 25% of all new HIV infections in the region (1). Oral pre-exposure prophylaxis (PrEP) for HIV prevention is recommended by the World Health Organisation (WHO) as a once daily pill for populations at high risk for HIV acquisition since 2015 (2) and in 2021, more than half of the countries in Eastern and Southern Africa were providing free oral PrEP to individuals at high risk of acquiring HIV, including AGYW (3). Provision of oral PrEP in these countries has largely been made possible under the auspices of the USA President’s Emergency Plan for AIDS Relief (PEPFAR), the Determined, Resilient, Empowered, AIDS-free, Mentored, and Safe (DREAMS) initiative, the Bill & Melinda Gates Foundation and the Global Fund for AIDS, TB and Malaria (4).

Despite the availability of oral PrEP in Eastern and Southern Africa, uptake and adherence to oral PrEP among AGYW are low (5, 6). Previous studies in Africa have indicated that barriers to oral PrEP uptake and adherence among AGYW may exist at various levels, including the individual (e.g., HIV-related stigma of HIV, fear of side effects, low PrEP awareness), interpersonal (e.g., parental influences, absence of a stable sexual partner), community (e.g., peer influence, social stigma around sexual behavior), institutional (e.g., long clinic waiting times, poor health worker attitudes), and structural level (e.g., cost of PrEP, mode of delivery, transport costs to access PrEP) (7, 8). Another possible reason for the low uptake and adherence to oral PrEP among AGYW in Eastern and Southern Africa is that AGYW may have diverse preferences and needs regarding PrEP that are not sufficiently addressed by oral PrEP. Indeed, some studies from this region have indicated that young people may prefer PrEP products with longer duration of protection than oral PrEP (9–12) which also ensure high adherence. A previous study among AGYW in Kampala, Uganda, showed that a reported preference for oral PrEP over other PrEP methods was associated with higher oral PrEP uptake (5). However, little is known about preferences for oral PrEP and other PrEP methods among AGYW in Uganda and other countries of Eastern and Southern Africa, and how these might be influenced by individual socio-demographic and reproductive health factors, sexual risk behavior, perceived HIV risk and substance use.

Long acting injectable cabotegravir (CAB-LA) given every 2 months and the monthly dapivirine vaginal ring have shown acceptable safety profiles and efficacy in clinical trials in Africa (13, 14) and these two methods alongside oral PrEP are recommended for use as PrEP by WHO (15–17). PrEP methods which require less frequent dosing and are more discreet than oral PrEP will likely improve adherence to PrEP but are not yet widely available and still under development. PrEP products in development include the biodegradable tenofovir alafenamide and cabotegravir reservoir PrEP implants (18, 19), broadly neutralizing antibodies (20, 21) and self-administered microarray patches (22). Furthermore, there is hope that lessons learned from HIV vaccine trials recently terminated due to futility (23, 24) as well as the advances of mRNA vaccine technology will promote future HIV vaccine development. The method mix of PrEP products is promising as it is expected to increase HIV prevention options for populations at high risk of HIV acquisition, such as AGYW. However, to the best of our knowledge, only one study, a qualitative study among 48 adolescents and adults, and 8 health workers in South Africa (25) has assessed AGYW’s preferences for a range of PrEP methods, including HIV vaccines. Yet, such information is crucial since it may inform PrEP product developers and the healthcare services about which methods have a higher chance of uptake and adherence among AGYW hence enhancing HIV prevention in this high-risk group.

We used a mixed methods study design including quantitative survey data from a cohort of 285 AGYW in Kampala, Uganda, to assess preferences for five biomedical HIV PrEP methods and qualitative interview data collected from a selected subset in the same cohort to explore reasons for PrEP method preferences and suggestions to make PrEP methods more appealing for use in this group.

## Materials and methods

### Study design

Between January and October 2019, we conducted a mixed methods study using a convergent parallel design (26). Quantitative data were collected through a cross sectional PrEP preference survey among a cohort of 265 AGYW aged 14–24 years in Kampala, Uganda. Trained study staff collected the data using interviewer administered questionnaires and we complemented this with qualitative semi-structured in-depth interviews with 20 selected study participants.

### Study setting

The study was conducted at the Good Health for Women Project (GHWP) clinic in Kampala, Uganda. The GHWP clinic was originally established in 2008 to conduct research on HIV and sexually transmitted infections (STIs) specifically among women involved in high-risk sexual behavior, including female sex workers (FSWs) (27). The clinic later provided health care services including HIV prevention, care and treatment, and sexual and reproductive health services to women and their regular male partners and conducted research among AGYW until December 2020 when it was closed. Health care services provided to participants during the current study included: HIV testing and counselling, male condoms, STI screening and treatment, contraceptives (oral, injectable and implants), pregnancy testing, and hepatitis B vaccination if naïve to hepatitis B or exposed but not immune. Oral PrEP was also offered to all AGYW. Laboratory tests were performed as part of routine health care services provided during the study.

### Study population, sampling, and eligibility

Participants in the current study were included from a cohort 285 AGYW who were enrolled at the GHWP clinic. The main aim of the cohort was to “assess knowledge and preferences for biomedical HIV prevention methods and uptake of oral PrEP among AGYW at high risk for HIV acquisition in Kampala, Uganda.”

#### Identification and recruitment for the AGYW cohort

Project field workers recruited AGYW peer leaders from urban slums characterized by entertainment facilities, where sex work, alcohol and illicit drug use were common. From January to October 2019, the field workers together with the peer leaders mobilized potential study participants from 22 communities in southern (10) and northern (12) Kampala located within the catchment area of the GHWP clinic. Participants were pre-screened to ascertain that minors (14–17 years) were emancipated/mature minors, who could legally consent to participate in research as per national guidelines (28). AGYW were enrolled in the cohort based on the following inclusion criteria: aged 14–24 years, HIV negative and at risk of HIV infection as shown by being sexually active in the past 3 months, living or working in sex-work hotspots in and around Kampala and willing to return for study follow up visit. They were excluded based on the following criteria: confirmed HIV infection, confirmed pregnancy, allergy to any substance and any uncontrolled acute or chronic infection. Details of the recruitment process and enrolment of emancipated/mature minors have been described previously (5).

#### Enrolment into the current study

Eligible cohort participants were enrolled and thereafter attended two education sessions on five biomedical PrEP methods, including oral PrEP, injectable PrEP, vaginal ring, PrEP implant and HIV vaccine(s). Of the 285 participants enrolled in the AGYW cohort, 20 participants did not return to the clinic after enrolment, they declined invitations to continue study visits and were excluded from the study, leaving a final analytical sample of 265 AGWY who attended the two education sessions.

##### Education on the five biomedical PrEP methods

Trained research nurses gave study participants their first education session on the five PrEP methods at enrolment and the second session within at least 2 weeks of enrolment. The five methods were chosen for inclusion since they have been assessed in pre-clinical studies or clinical trials and included those that were already available for use *(oral PrEP)*, likely to be available soon *(injectable PrEP and vaginal ring)*, and those still in development *(PrEP implant and HIV vaccines)*. Information provided by the nurses included the method’s mode of delivery (product form), dosing frequency, known or potential side effects, actual demonstration of available samples, e.g., oral PrEP and vaginal ring, and use of licensed vaccines or contraceptive proxies to demonstrate other methods. Information also included whether products were available for use or still in development. Education sessions were conducted according to a protocol developed by the study team and sponsor, to ensure that staff administered it in a standardized way (Supplementary File S1). The protocol was translated to the local language (Luganda) and included visualization (pictures) of the PrEP methods. After the second session was completed, participants were assessed on their understanding of the five PrEP methods through an interviewer administered 5-item questionnaire (one question on each method) and study staff clarified when a method had not been understood (Supplementary File S2). They then responded to the preference survey regarding the five PrEP methods. Table 1 shows a summary of information given during the two education sessions.

**Table 1:** Summary of the information given to the study participants during the two education sessions and before assessing their preferences regarding PrEP methods.

**PrEP method or product**	**Product form and mode of delivery**	**Dosing frequency**	**Documented side effects at the time of the study**	**Product demonstration**	Product availability globally at the time of the study
Oral PrEP	Pills, swallowed	Daily	Nausea, vomiting, stomach-ache, headache, muscle pain	Actual oral PrEP pills	Available
Injectable PrEP	Injection in the muscle	2–3 months	Sleep problems, mild nausea, vomiting, stomach pain, diarrhoea, headache, dizziness, fever, fatigue, flu-like illness, mild skin rash, allergy, injection site reactions	Drug Vial and injection	Not available
Vaginal Ring	Flexible ring, Vaginal insertion	Monthly	Irregular bleeding between menstruation periods, reduced frequency of menstruation reddening, or swelling of the opening of the uterus. Urinary tract infection, candidiasis, itching of vulval skin, headache; pain during sex, pelvic pain	Actual vaginal ring	Not available
PrEP Implant	Implant, inserted under the skin	12 months	Pain and swelling at the site of insertion.	Contraceptive implant as proxy	Not available
HIV Vaccine	Injection on the upper arm	3–4 doses over 6 months, thereafter lifetime protection	Reactions at the injection site such as pain, swelling, itching, soreness.	Hepatitis B vaccine vial and injection as proxy	Not available

### Laboratory methods

Serum and endo-cervical swabs were collected by trained research nurses. Trained laboratory technologists performed tests on serum for HIV (Determine screening test, Statpak confirmatory test, SD Bioline as tiebreaker). Chlamydia and gonorrhoea tests were performed on endo-cervical swabs using GeneXpert (Cepheid AB, Solna Sweden).

### Quantitative data on PrEP preferences and participants’ characteristics

During the same study visit as the preference survey, trained research nurses used interviewer administered questionnaires to collect data on socio-demographic factors, sexual behavior, substance use, HIV status of sexual partner(s) and PrEP method preference, described in detail below under ‘main outcome variable’. All data were double entered in Open Clinica.

### Main outcome variable

The main outcome was *“*Participants’ most preferred PrEP method.”

After the two education sessions on the five PrEP methods, participants were asked the following question:

*“Of the 5 methods mentioned (daily oral pills, 2–3 monthly injections, monthly vaginal ring, implant inserted in the arm for a year and 3–4 doses of an HIV vaccine), if all would be available, mention which ones you would be willing to use in order of preference. Please give your preference even if the method is not yet available.”*

Volunteers ranked their preference of the five methods on a scale of 1 to 5 (1 = “Most preferred,” 5 = “least preferred”).

### Exposure variables

#### Socio-demographic factors

Age at enrolment in years; marital status (married, separated/divorced, single/never married); educational level (none, primary, secondary, tertiary), main job (sex work; hospitality, e.g., working in a restaurant, massage parlor, hair salon; entertainment, e.g., working in a bar/night club, karaoke venue; no job; other, e.g., market vendor, mobile street vendor, cleaner); and number of biological children.

#### Substance use

Alcohol use in the past 12 months was assessed using a 10-item questionnaire, the Alcohol Use Disorder Identification Tool/AUDIT (29). The Audit scores for the responses were summed up and categorized as “low to moderate risk” drinking (0–15) and “high-risk” drinking (≥16). We also assessed drug use in the past month (Yes/No). Drugs were categorized as khat, injection drugs, marijuana and others.

#### Sexual behavior and reproductive health factors

Participants were assessed for the number of sexual partners in the past 3 months (<10, ≥10); condom use with sexual partners in the past 3 months (Yes/ No), frequent travel from home in the past 3 months, i.e., ≥3 nights away from home per week (Yes/ No), receiving money gifts or other favors for sex in the past 3 months (Yes/ No); reported anal sex, forced sex or group sex in the past 3 months (Yes/No); contraceptive use (hormonal and non-hormonal) in the past 3 months (not using, using a short acting method, i.e., pills condoms, using a long-acting method, i.e., injectable, implants, intra-uterine device, lactational amenorrhoea) and laboratory diagnosis of chlamydia and/or gonorrhoea (Yes/No).

Data were also obtained on HIV status of sexual partner(s) (Negative, Positive, Unknown).

### Other variables

#### Ever heard about biomedical PrEP methods

Participants were assessed if they had ever heard about the following methods before study enrolment; oral PrEP, injectable PrEP, the vaginal ring, PrEP implant and HIV vaccine(s).

#### PrEP method they would not be willing to use

Participants were also assessed for which methods they would not be willing to use at all if all were available.

### Statistical analysis, quantitative data

All analyses were conducted with STATA 17.0 (StataCorp, College Station, TX, USA). Characteristics of the study participants were summarized as descriptive statistics using frequencies and percentages. Preference for each PrEP method was determined as the direct proportion of participants who preferred that method over the total number of participants assessed for method preference and is presented as proportions with 95% confidence intervals (CI). The analysis was based on preference being a relative measure where a participant prefers one PrEP method over other options. Participant’s “most preferred” method was considered, and a four level polytomous outcome variable was generated with “oral PrEP most preferred” as the reference category. Daily oral prep was considered as the reference category given that oral PrEP was the only method available for use at the time of the study. The other 3 outcome categories were “injectable PrEP most preferred,” “PrEP implant most preferred” and “HIV vaccine most preferred.” Given the small proportion that preferred the vaginal ring (*n* = 3, 1.1%), this method was not included in the polytomous dependent variable of most preferred biomedical PrEP method. Each exposure was cross-tabulated with the outcome, and exposures for which some categories had no participants with the outcome were not considered in the modelling. Hence, the variable “anal sex in the past 3 months” was dropped because of those reporting anal sex, none preferred the PrEP implant. Multinomial logistic regression was used to determine associations of exposures [socio-demographic factors, substance use, sexual behavior factors and HIV status of partner(s)] with most preferred biomedical PrEP methods. All exposures were treated as categorical variables except for age which was analyzed as a continuous variable. At the unadjusted analysis, each exposure was assessed with the main outcome and only those for which the associations attained statistical significance at *p* = 0.15 were considered for the adjusted multinomial logistic regression model. At adjusted analysis, factors were removed from the model if they were not significant in any of the preferred method categories and if removing them did not make the model fit significantly worse at *p* = 0.05 on a likelihood ratio test (LRT). Therefore 3 variables which were not significant at adjusted analysis and did not significantly improve the model on the LRT were removed from the final model, i.e., “number of biological children,” “frequent travel from home in the past 3 months” and “use of family planning methods.” The final multivariable model was adjusted for age, number of sexual partners in the past 3 months, paid sex in the past 3 months, chlamydia and/or gonorrhoea diagnosis and HIV status of sexual partner(s). Unadjusted relative risk ratios (uRRR) and adjusted relative risk ratios (aRRR), 95% CI, and *p*-values are reported. All results with a p-value of <0.05 were considered significant.

### Qualitative data collection and analysis

Approximately 10% of enrolled participants were purposively sampled for inclusion in IDIs to obtain data on preferences according to two exploratory aims, i.e., reasons for their preferences for certain PrEP methods and suggestions of how to make products more appealing to young people. Participants were selected from the database according to the PrEP method they had chosen as “most preferred” and initially placed in six categories of “most preferred method” as follows: oral PrEP (5), injectable PrEP (5), PrEP implant (5), HIV vaccine (5), vaginal ring (5) and “No preference” (5), but the latter two categories (vaginal ring vs. no preference) were selected by too few respondents to make any IDIs meaningful, settling the final number of IDIs at 20 across the first four methods. The participants were contacted by phone using the contact they gave to the field worker. IDIs were scheduled at the study clinic with an option of another place of the participant’s choice. Two trained female research assistants took notes and audio recorded IDIs conducted using a semi-structured interview topic guide that had been piloted beforehand and translated to Luganda, the well understood local language. IDIs were conducted within 1–15 days after the second education session and took between 45 and 60 min. The following topics were explored: (i) factors influencing method preference (e.g., individual factors such as prior experiences with products having similar mode of delivery, product attributes); (ii) alternative preferences to their most preferred method and methods they would not use at all; (iii) suggestions to improve method appeal. Audio-recordings from IDIs were transcribed verbatim, translated verbatim to English and coded. An initial list of codes was generated by coding 4 transcripts through a process that was both inductive and deductive. Initial codes were refined and organized by 2 analysts into a coding framework which was used to code the entire data set. The analysts completed coding of the transcripts using NVivo 14, adding updates to the coding framework. Thematic analysis was used to organize and analyze the dataset. The qualitative data had equal weighting as quantitative data during interpretation of results.

### Ethical considerations

Before study start, the study was approved by the Uganda National Council for Science and Technology (HS 2435) and Uganda Virus Research Institute-Research Ethics Committee (GC/127/18/06/658). Written informed consent was obtained from all participants before data collection, including consent for audio-recording of IDIs. Confidentiality was maintained by use of numerical identifiers on all participant’s data and samples. Documents with personal identifiers were locked and only accessed by the principal investigator and their designee.

## Results

### Characteristics of study participants (Table 2)

Table 2 shows the characteristics of the 265 AGYW included in the study. Mean age was 20 years (SD ± 2.2), 61.5% were ≥ 20 years, 55.1% had attained secondary level education or higher, 64.5% had at least one biological child and 57.3% were single (never married). The prevalence of drug use (any drug) in the past month was 16.2, and 14.7% were assessed as high-risk alcohol drinkers. All 265 participants reported that they were sexually active with mean age at sexual debut being 15.7 years (SD ± 2.1). Overall, 92.8% reported engaging in paid sex in the past 3 months, however only 21.9% self-identified as sex workers. Participants who reported having no job were 24.9% (*n* = 66) of whom 89.4% reported engaging in paid sex in the past 3 months. A total of 54 (20.4%) participants reported ≥10 sexual partners in the past 3 months, of whom 66.7% self-identified as sex workers or reported no job while 33.3% had other jobs. The prevalence of STIs (chlamydia and/or gonorrhea) at baseline was 25.8%.

**Table 2:** Characteristics of the 265 AGYW study participants who were assessed for preferred biomedical PrEP methods in Kampala, Uganda, in year 2019.

**Characteristic**		**Number, *n***	**Percentage (%)**
Socio-demographics			
Participant’s age group at enrolment			
	Adolescents (14–19 years)	102	38.5
	Young Women (20–24 years)	163	61.5
Education level of participant			
	Less than secondary	119	44.9
	Secondary or higher	146	55.1
Marital status of participant			
	Single (never married)	153	57.7
	Married	75	28.3
	Separated/ Divorced	37	14.0
Number of biological children			
	None	95	35.9
	One or more	170	64.2
Main occupation			
	Hospitality/ Entertainment/ Other	141	53.2
	No job/ Sex work	124	46.8
Sexual behavior and reproductive health			
Total number of sexual partners in past 3 months			
	Less than 10	211	79.6
	10 or more	54	20.4
Received payment for sex in the past 3 months			
	Yes	246	92.8
Experienced forced sex in the past 3 months			
	Yes	62	23.4
Travelled frequently from home in the past 3 months			
	Yes	110	41.5
Reported condom use with sexual partners			
	Yes	194	73.2
Had CT and/ or NG diagnosis †			
	Yes	68	25.8
Using a family planning method			
	Not using	120	45.3
	Using a short-acting method	47	17.7
	Using a long-acting method	98	37.0
**Substance use**			
Alcohol use using AUDIT Tool			
	Low to moderate risk drinking	226	85.3
	High risk drinking	39	14.7
Drug use in the past 3 months			
	Yes	43	16.2
HIV status of sexual partner(s)			
	HIV Negative	45	17.0
	Living with HIV of Unknown	220	83.0
Other			
Baseline knowledge of oral PrEP as an HIV prevention method			
	Know about oral PrEP for HIV prevention	65	24.5
	No knowledge about oral PrEP	200	75.5

^†^One volunteer not screened for Sexually Transmitted Infection (STIs).

Of 145 (54.5%) participants using both hormonal and non-hormonal contraceptive methods, the most common methods were injectables (31.0%) implants (31.0%) and condoms (24.8%).

The proportion of study participants that had ever heard about each prevention method prior to study enrolment was 24.2% for oral PrEP, 4.2% for injectable PrEP, 2.3% for vaginal ring (2.3%) and 1.5% for HIV vaccine (1.5%), respectively. No participant had ever heard about the PrEP implant.

### Preference for biomedical PrEP methods (Figure 1)

**Figure 1:**
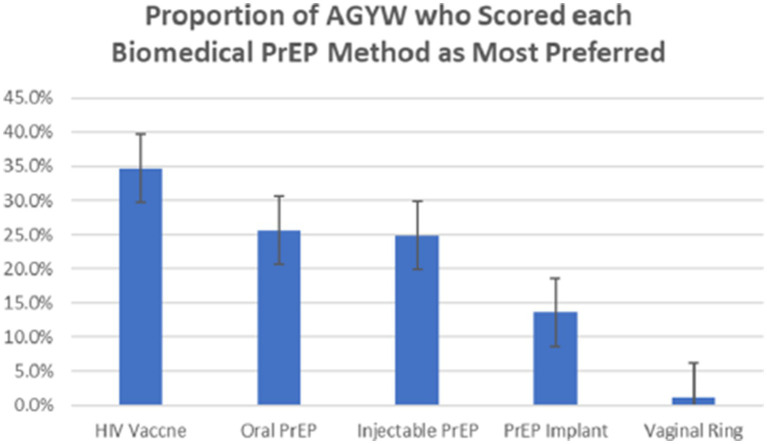
Proportion (% and corresponding 95% CI) of AGYW study participants who scored each biomedical PrEP method as their most preferred, *n* = 265, Kampala, Uganda (2019).

After receiving two educational sessions, participants scored their *“most preferred”* PrEP methods, as illustrated in Figure 1. HIV vaccine was reported as the most preferred PrEP by 34.7% (95% CI 29.1–40.7%) of study participants, oral PrEP by 25.7% (95% CI 20.7–31.3%), injectable PrEP by 24.9% (95% CI 20.0–30.5%), PrEP implant by 13.6% (95% CI 9.9–18.3%) and vaginal ring by only 1.1% (95% CI 0.4–3.5%), respectively. Considering the average score of each PrEP method across all participants, average scores were: HIV vaccine (2.36), oral PrEP (2.67), injectable PrEP (2.40), PrEP implant (3.01) and vaginal ring (4.43). The HIV vaccine had the lowest average closest to 1 “most preferred method” while the vaginal ring had the highest average closet to 5 “least preferred method.” The 81 AGYW who started using oral PrEP during the study, ranked this method as follows: rank 1 “most preferred” (n = 27), rank 2 “preferred” (n = 23), rank 3 “moderately preferred” (n = 16), rank 4 “slightly preferred” (n = 10) and rank 5 “least preferred” (n = 5). Thus, a proportion of AGYW took up oral PrEP not because it was their most preferred method, but because it was the only biomedical PrEP method available.

When asked which method they would not be willing to use at all if all were available, 64.2%; (95% CI 58.2–69.7%) mentioned the vaginal ring.

### Multinomial logistic regression for the association between characteristics of the AGYW and preferences for PrEP method (Table 3)

**Table 3:** Multinomial Logistic Regression for the association between characteristics of the 265 AGYW study participants’ and their most preferred PrEP method.

		**Injectable PrEP**				**PrEP implant**				**HIV vaccine**			
**Characteristic**	**Category**	**uRRR (95%CI)**	***p*-value**	**aRRR (95%CI)**	***p*-value**	**uRRR (95%CI)**	***p*-value**	**aRRR (95%CI)**	***p*-value**	**uRRR (95%CI)**	***p*-value**	**aRRR (95%CI)**	***p*-value**
Age at enrolment (years)		1.21 (1.03–1.43)	0.021	1.22 (1.04–1.44)	**0.018**	1.37 (1.11–1.69)	0.003	1.42 (1.14–1.77)	**0.002**	0.99 (0.86–1.14)	0.908	0.99 (0.86–1.14)	0.845
Number of biological children													
	None	Ref				Ref				Ref			
	One or more	1.87 (0.90–3.86)	0.092			2.90 (1.11–7.55)	0.029			0.87 (0.46–1.64)	0.669		
Number of sexual partners in past 3 months													
	Less than 10	Ref		Ref		Ref		Ref		Ref		Ref	
	10 or more	1.04 (0.42–2.59)	0.939	1.19 (0.46–3.06)	0.717	2.59 (1.01–6.68)	0.049	3.14 (1.16–8.55)	**0.025**	1.35 (0.59–3.06)	0.474	1.52 (0.65–3.54)	0.332
Paid sex in the past 3 months													
	No	Ref				Ref				Ref			
	Yes	0.23 (0.03–2.13)	0.196	0.36 (0.04–3.51)	0.377	0.52 (0.03–8.61)	0.650	0.78 (0.04–14.24)	0.865	0.10 (0.01–0.79)	0.029	0.12 (0.02–1.01)	0.052
Frequent travel from home in past 3 months													
	No	Ref				Ref				Ref			
	Yes	1.20 (0.61–2.37)	0.599			2.00 (0.86–4.63)	0.106			1.71 (0.90–3.22)	0.100		
Had Chlamydia/Gonorrhea													
	No	Ref		Ref		Ref		Ref		Ref		Ref	
	Yes	2.42 (1.06–5.53)	0.037	2.53 (1.08–5.90)	**0.032**	2.59 (1.01–6.68)	0.049	2.66 (0.99–7.11)	0.052	1.75 (0.79–3.90)	0.169	1.65 (0.72–3.75)	0.234
HIV status of sexual partner(s)													
	HIV negative	Ref				Ref				Ref			
	Unknown/HIV positive	0.29 (0.01–0.87)	0.027	0.30 (0.10–0.91)	**0.034**	0.33 (0.10–1.12)	0.076	0.25 (0.07–0.92)	**0.037**	0.30 (0.11–0.86)	0.025	0.37 (0.12–1.09)	0.072
Use of family planning methods													
	Not using	Ref				Ref				Ref			
	Short acting	0.93 (0.36–2.40)	0.874			1.50 (0.46–4.91)	0.503			0.92 (0.40–2.12)	0.852		
	Long acting	1.98 (0.92–4.26)	0.081			3.68 (1.44–9.45)	**0.007**			1.16 (0.56–2.40)	0.687		

Reference category, oral PrEP most preferred; uRRR, unadjusted Relative risk ratio; aRRR, adjusted relative risk ratio; CI, confidence intervals; Bold values indicate statistical significance (*p* < 0.05). The final multivariable model was adjusted for age, number of sexual partners in the past 3 months, paid sex in the past 3 months, chlamydia and/or gonorrhoea diagnosis and HIV status of sexual partner(s).

When compared to the reference category (oral PrEP most preferred) the likelihood of injectable PrEP being most preferred increased by 22% for every one-year increase in age (aRRR 1.22; 95% CI 1.04–1.44). Preference for injectable PrEP was more likely among those with a diagnosis of chlamydia and/ or gonorrhoea compared to those without (aRRR 2.53; 95% CI 1.08–5.90) and less likely among those having sexual partner(s) living with HIV or of unknown HIV status compared to those living with a known HIV negative partner (aRRR 0.30; 95% CI 0.10–0.91). The likelihood of PrEP implants being most preferred increased by 42% with every one-year increase in age (aRRR 1.42; 95% CI 1.14–1.77), was higher among those with ≥10 sexual partners in the past 3 months compared to those with <10 sexual partners while it was lower among those having sexual partner(s) living with HIV or of unknown HIV status compared to those living with a known HIV negative partner (aRRR 0.25; 95% CI 0.07–0.92). The association with chlamydia and/or gonorrhoea diagnosis at baseline achieved borderline significance (aRRR 2.66; 95% CI 0.99–7.11). Associations of exposures with preference for an HIV vaccine achieved borderline significance for living with sexual partner(s) living with HIV or of unknown HIV status compared to living with a known HIV negative partner (aRRR 0.37; 95% CI 0.12–1.09) and reporting paid sex in the past 3 months compared to not reporting paid sex (aRRR 0.12; 95% CI 0.02–1.01).

### Results of qualitative data analysis (summarized in Table 4)

**Table 4:** Summary of reasons influencing preference for biomedical PrEP methods among AGYW invited for IDIs in Kampala, Uganda (2019–2020) *n* = 20.

**Most preferred/alternative method**	**Reasons facilitating method preference**	**Reasons hindering method preference**
Oral PrEP	Ease of use (just swallow a pill), non-invasive, not painful, short duration (in case of side effects)	Pill burden, non-discreet, similar appearance and packaging to ARVs, forgetting to swallow daily pills, prior negative experience with other oral drugs, e.g., smell of drugs, nausea
Injectable PrEP	Longer duration of protection, discreet use, familiarity with injectable contraception	Invasiveness, pain
PrEP implant	Longer duration of protection, discreet use, familiarity with contraceptive implant	Invasiveness, pain, prior negative experience with scarring of the skin/keloids
HIV vaccine	Completion of doses with protection for life	Invasiveness, pain, vaccine induced sero-positivity.
Vaginal ring	*No IDIs conducted (Very few participants preferred the vaginal ring)*	Insertion (pain, wrong insertion, unable to clean during menses), STIs, 1 month duration short; partner will feel it, perception that ring is too big *Reasons given by participants who did not prefer the vaginal ring but compared their preferred method to the vaginal ring.*

Twenty IDIs were conducted with participants who preferred oral PrEP (5), injectable PrEP (5), PrEP implant (5) and HIV vaccine (5). We observed that participants had understood and could remember the information they received during the two education sessions as shown in Supplementary File S3.

The IDIs explored two main exploratory aims (i) reasons for preference of PrEP products (oral PrEP, long-acting PrEP products) (ii) suggestions for improving product appeal.

#### Factors influencing preference for oral PrEP

Five participants who preferred oral PrEP reported that they were positively influenced by product attributes, e.g., ease of use (swallowing), pills being a common and familiar method used in health care and its mode of administration being neither painful nor invasive. One participant described how she liked the short duration of action of oral PrEP because the drug would be eliminated from the body quicker than an injected drug for which it would be more difficult to manage any adverse side effects to the drug.

“I have to first use this one [oral PrEP]. It is the one that is easy for me. Because I don’t know what problems I would get with it [injectable PrEP] and there is no way that the drug would be removed from my body [in case of a drug reaction]…”

(Sex worker, 20-24 years, secondary education or higher, preferred oral PrEP).

A younger participant who reported having no job but engaged in paid sex, also described the easy mode of administration which was an attribute that she appreciated about oral PrEP.

“… Because with pills it is easy. … I don’t want my body to be pierced repeatedly. Now for example these other things [PrEP implant], I don’t want to put them inside me.”

(No job, 14-19-years, living with boyfriend, secondary education or higher, preferred oral PrEP)

Other attributes of oral PrEP negatively influenced preference, e.g., pill burden, pill containers not being discreet, stigma due to use of oral anti-retroviral drugs (ARVs) for both treatment and prevention of HIV and prior individual experiences like side effects with pills used for other illnesses. Excerpts from two interviews in which concerns about pill burden and oral PrEP being similar to oral ARVs are shown below.

“We can swallow them [oral PrEP pills], but not daily, because you can get tired of them. It is better to take pills after every few days, but every day! As if you are living with HIV”.

(Market vendor, 14-19 years, secondary education or higher, preferred an HIV vaccine).

“I also saw they [Oral PrEP] look like the drugs for HIV. So someone can think you are taking ARVs and then he or she gets worried. That is why I don’t want the pills. It is better if it [PrEP] is inside me and they don’t see it”.

(Sex worker, 20-24 years, less than secondary education, preferred a PrEP Implant).

#### Factors influencing preference for long-acting methods

Fifteen participants who preferred injectable PrEP, the PrEP implant or an HIV vaccine were positively influenced by product attributes like discreetness, longer duration of protection or protection for life, and familiarity with contraceptive methods having similar mode of delivery. In the conversation below, a participant describes how the lifelong protection offered by an HIV vaccine influenced her preference.

RES: The one which I prefer is still being researched about, it is the vaccine.

INT: Why the vaccine?

RES: They tell us that the injection for vaccinating is given to you for example 3-4 doses and you get protection for the rest of your life … That injection is good because they say that it is for your lifetime but for PrEP, I would have to come to the clinic to get it every month. You swallow it continuously.

(Sex worker, 20-24 years, less than secondary education, preferred an HIV vaccine).

Another participant described how she preferred injectable PrEP due to familiarity with the injectable contraceptive.

“For that one [Injectable PrEP], since I am using family planning, it is easy for me because it is given like family planning. For example, when my family planning method reaches 3 months, I go back, and they inject me with another dose. So, it is the same routine as the injection for family planning, that is why I have chosen it.”

(Market vendor, 14–19 years, secondary education or higher, preferred injectable PrEP).

A few participants did not like the invasiveness and pain associated with injections, and one participant was concerned about vaccine-induced seropositivity, and worried that she would have to explain every time if she got an HIV test away from the study site.

“Then there is also the other one which vaccinates. For that one if they test you [for HIV] it may seem like you have HIV even if you don’t have it. If that health worker tests you and you didn’t tell him or her that you were vaccinated [HIV vaccine], they wouldn’t understand it. … because the health worker explained it to us. If you go to another health facility to get tested for HIV, you may not be able to explain and they believe you …”

(Sex worker, 20-24 years, less than secondary education, preferred a PrEP implant)

It is important to note that due to limited options available at the time of the study, some participants who preferred long-acting methods started oral PrEP as cited below.

“Yes, they are not available that is why right now I am taking PrEP [Oral PrEP]. You know you have to try out everything. I am using PrEP right now but if these other products become available, that is when I will start using the implant.”

(Works in a hair salon, 20-24 years, secondary education or higher preferred a PrEP Implant).

When asked for alternative choices to their most preferred PrEP method, majority of IDI participants mentioned injectable PrEP or an HIV vaccine while few mentioned oral PrEP.

We observed that the two most common factors influencing preference for long-acting PrEP methods (Injectable, implants and vaccine) were longer duration of protection or protection for life and discreetness during use. Those for whom oral PrEP was an alternative PrEP method mentioned that pills were easy to swallow however, pill burden and similarity to oral ARVs were common barriers against oral PrEP preference.

#### Suggestions to make PrEP methods more appealing to AGYW

We present data from 14 participants’ who gave their perceptions on how to improve PrEP appeal. We observed 3 themes: (i) suggestions for product alteration, e.g., smaller pill and vaginal ring, longer acting pill and vaginal ring; (ii) suggestions for community education and assurance of product safety and (iii) suggestions to increase PrEP options that meet individual preferences. Two participants are quoted below giving suggestions about product alteration and education, respectively.

“That one [vaginal ring], they can change it. They will decide how to improve it. I think they should also reduce its size; it is too big. By the time they put it inside you, you are already scared.

(Sex worker, 20–24 years, less than secondary education, preferred injectable PrEP).”

“Everyone is supposed to choose for themselves what they want to use. So, health workers would have to educate people [in the future] and teach them how these methods work. More people would then learn the methods and choose what they want to use.”

(Works in a restaurant, 20-24 years, less than secondary education, preferred a PrEP implant).

## Discussion

This mixed methods study assessed preferences for both available biomedical PrEP methods and those under development among AGYW at high risk of HIV acquisition in urban Kampala, Uganda. Preferences among AGYW for the different PrEP methods varied, with higher preference (73% of the study participants) for long-acting methods. Common reasons for preference for long-acting methods as shown by our qualitative data were long duration of protection, discreetness, and individual experiences, e.g., familiarity with injectable and implantable contraceptives as previously documented among AGYW (7, 8) and other female populations in sub–Saharan Africa (30). When compared to oral PrEP, preference for long-acting methods was higher with increasing age and among those with higher sexual behavior risk (higher number of sexual partners, STIs), while it was lower among those with sexual partners living with HIV or with unknown HIV status.

Previous studies among young people in Eastern and Southern Africa have also reported higher preference for long-acting PrEP methods (9, 12, 25, 31). It is therefore not surprising that AGYW in the current study suggested that a longer acting pill and vaginal ring would be more appealing, as accessibility of methods and compliance make daily or frequently administered regimens challenging in this population of AGYW. High acceptability for a 3-month vaginal ring has been reported among women outside sub–Saharan Africa (32). There is also hope that oral PrEP will have long-acting options. For example, initial trials of long-acting pills for both PrEP and HIV treatment have proposed a weekly and monthly pill which are both attractive as they deal with the pill burden and stringent adherence schedule of daily oral PrEP. Participants who received Islatravir-containing regimens experienced a dose-dependent drop in CD4 and total lymphocyte cell counts leading to halting of the trials (33). However, newer and improved long-acting pills continue to be tested alongside other long-acting formulations of Islatravir, i.e., the sub-dermal implant (34) and will offer more PrEP options for AGYW. Regarding adherence to oral PrEP, an analysis of data from *cis*-gender women in Eastern and Southern Africa (4 countries), India and the United States identified four distinct oral PrEP adherence patterns that corresponded to different levels of protection as shown by the HIV incidence estimates. Two of these groups, “consistent daily” (7 doses/week) and “consistently high” (4–6 doses/week) were both associated with very low HIV incidence while the “high but declining” (4–6 doses/week and then declined), and “consistently low” (<2 doses/week) were associated with higher HIV incidence (35). The “consistently high” pattern of adherence identified by Marazzo et al. reduces the pill burden for women while still maintaining high HIV protection rates. This finding will reposition the discussion around oral PrEP dosing for cis-gender women with on-demand regimens being tested and recommended if efficacy is demonstrated. Even though majority of participants preferred long acting methods, daily oral PrEP currently still has a place in HIV prevention given that it is the most available method and most preferred for a quarter of AGYW, and we have previously demonstrated that higher oral PrEP uptake among AGYW is associated with preference for oral PrEP (5). Modification of oral PrEP attributes, e.g., on-demand regimens, long-acting pills and less than daily dosing options for those with continued sexual exposure will likely influence preference and subsequent uptake of oral PrEP in future.

As suggested by IDI participants, increasing options to cater for different preferences will make methods more appealing to these young women. Furthermore, addition of more long-acting PrEP methods will normalize HIV prevention as products (available and in-development) are like prevention and treatment methods already used in health care. These IDI findings are timely given the recent WHO conditional recommendation for the dapivirine ring as an additional prevention choice for women at high risk of HIV acquisition and the WHO recommendation to include long-acting injectable cabotegravir (CAB-LA) as an additional PrEP option for individuals at high risk of HIV (17). The risk of developing drug resistance to cabotegravir and other integrase inhibitors as has been reported in both pre-clinical and clinical studies (36–38) is an important consideration. This risk continues to exist among users of CAB-LA, due to its long pharmacokinetic tail, of concern particularly for individuals who acquire HIV after they stop the drug but have remaining residues of sub-optimal ARVs for several months (39). Smith et al. have modelled the impact of introduction of CAB-LA in Sub-Sharan Africa over a 20-year period and report that drug resistance will certainly increase. However there will be significant benefits in terms of increased PrEP use, reduced HIV incidence, reduced AIDS deaths and similar cost effectiveness as oral PrEP delivery if CAB-LA is delivered at the same cost as oral PrEP and use of antibody rapid tests is maintained (39). An HIV vaccine(s), one of the anticipated long-acting options, had the biggest proportion of AGYW who scored it as their most preferred method. The RV144 HIV vaccine trial that used a prime-boost regimen given in 6 months is the only vaccine trial that has shown some efficacy (31%) todate. The trial reported rapid decline of initially high immune responses however, late boosting of the RV144 regimen shows that efficacy may be improved with longer intervals between the primary vaccination series and late booster dose (40). It is important to note that in future, an HIV vaccine(s) that needs regular booster doses may require similar or more effort from the end-user than an individual using injectable PrEP or a PrEP implant in light of on-going improvements with other PrEP methods. For example, a new drug, injectable (sub-cutaneous) lenacapavir given 6-monthly with the advantage that it could be self-administered will soon be tested in Uganda and South Africa (NCT04994509) (41). Longer duration of protection may therefore be a game changer for future HIV vaccine(s). Newer vaccine strategies focused on eliciting broadly neutralizing antibodies (42) and functional killer T-cells (43), are being employed to develop HIV vaccines that offer broad, durable responses that would ensure a profile that offers benefits over what has been achieved with long-acting PrEP products. After the rapid vaccine development during COVID, delivery technologies such as viral vectors and mRNA technology are being employed to accelerate the development timeline for HIV vaccines.

Majority of IDI participants mentioned the vaginal ring when asked which method they were not willing to use, mainly due to concerns around vaginal insertion, a finding similar to that of a study that assessed acceptability of the contraceptive vaginal ring among female adolescents (44). This corroborates findings of low preference for the vaginal ring as seen in two discrete choice experiments among AGYW in Kenya and South Africa (12, 45). Our findings could be explained by the fact that our participants have never interacted with this mode of drug delivery given that contraceptive vaginal rings are not available in the Ugandan setting. Additionally, only a few AGYW in Uganda may have participated in clinical trials that enrolled adult women to evaluate the dapivirine vaginal ring (14, 46). This however may not hinder the use of the ring once available, since studies from low and middle income countries show an increasing acceptability of the ring with familiarity of use (11, 47, 48) and, IDI participants also suggested that community education on products and their safety would improve method appeal.

We found a clear association between increasing age and a higher likelihood of AGYW choosing both injectable PrEP and the PrEP implant as their most preferred PrEP methods over oral PrEP, and especially for PrEP implants (37% vs. 21%) that would offer even longer duration of protection than the injection. Indeed, one of the factors influencing preference for the PrEP implant or injectable PrEP among IDI participants was familiarity with contraceptive methods having similar mode of delivery. Literature shows that older women are more likely to use contraception (49), likely because they already have a biological child (50, 51), which might have contributed to the observed association of preference for injectable PrEP or the PrEP implant with increasing age. Contraceptive use among unmarried adolescents and young women in our setting is still low, likely due to social norms that act as barriers (52, 53). Therefore, the older participants, who have experience with injectable and implantable contraceptives may be more likely to prefer PrEP methods having similar mode of administration. Nonetheless, future trials of the PrEP implant likely need to show similar or higher efficacy than CAB-LA to be approved.

Multi-purpose prevention technologies (MPTs) that prevent both HIV, possibly other STIs, and, unintended pregnancy, are also in the pipeline when developing biomedical PrEP methods and including contraception will likely make future PrEP products even more appealing. A qualitative survey of the PrEP Implant that included AGYW in Gauteng, South Africa indicates that 82% prefer a product with dual protection against HIV and unintended pregnancy (54). Furthermore, studies of MPTs combining PrEP and contraception, e.g., a qualitative study exploring perceptions of micro array patches in Kenya and a cross-over trial of placebo products (injection, tablet, ring) in South Africa have shown high acceptability for MPTs among AGYW and FSWs (11, 55).

Participants with ≥10 sexual partners in the past 3 months were 3 times more likely to prefer the PrEP implant when compared to those with fewer partners. Continued sexual exposure through multiple sexual partnerships means longer term vulnerability to HIV and likely explains their preference for the PrEP implant which would provide protection for several months. Studies in South Africa show that duration of action of PrEP methods is an important attribute for young people (10, 56). In a qualitative survey of a hypothetical PrEP implant, AGYW preferred a 12-months over a 6-month product (54). Long-acting methods have the advantage of discretion as highlighted in our results and, for this group of AGYW who frequently reported paid sex, it is important that preventive methods do not expose their sexual behavior practices. Similarly, participants with chlamydia and/or gonorrhea at baseline, also indicating high risk behavior (and possibly less frequent STI-screening or poorer health seeking behavior) were more likely to prefer injectable PrEP, but a similar association only achieved borderline significance for the PrEP implant. Among contraceptive users with an STI diagnosis in our study, the bigger proportion were using the injectable (almost one third) or contraceptive implant (over one third). STIs also indicate sexual risk behaviors and the choice of long-acting PrEP methods that are discreet is not surprising. An experimental study in South Africa suggests that STI protection is an important product attribute for FSWs in addition to protection against HIV and pregnancy (45). These findings further support MPTs whose prevention scope includes curable STIs.

When compared with participants having HIV negative sexual partner(s), those who had partners of unknown HIV status or partners living with HIV were less likely to prefer injectable PrEP or the PrEP implant. This is likely explained by higher risk perception given the more proximal risk of HIV acquisition from sexual partners and the need to protect themselves. Hence the lower preference for unavailable methods when compared to oral PrEP that was provided in the study. Findings from a registration cohort of FSWs enrolled in an HIV vaccine trial in Tanzania and the Partners Demonstration Project that integrated PrEP and ART delivery among HIV Sero-discordant couples in Kenya and Uganda showed higher PrEP use among women whose sexual partners were living with HIV (57, 58). Oral PrEP is an available user-controlled method and in the context of sex work, would provide cover in cases of failed condom negotiation or acts of sexual violence from clients of unknown HIV status (59–61). These results however may be different in a setting where all methods are available given the higher preference for long-acting methods that we report.

### Strengths and limitations

Firstly, our study was limited by the use of non-random sampling methods (which may lead to selection bias) to recruit and enroll participants, but the purpose was to recruit a study population that was representative of AGYW at high risk of HIV infection who represent potential future users of PrEP, rather than that of the general population of AGYW in the region. Secondly, products such as the PrEP implant and a potential future HIV vaccine are still in development stages, making it difficult to discuss characteristics like dose and regimen for such products with any certainty. For example, the information about an HIV vaccine providing lifetime protection is a major limitation as future vaccines will likely require repeated injections to be effective. Thus, our findings on preferences for products in development may not reflect actual preferences as final products become available with more or slightly different attributes than those we assessed. Nevertheless, our results are corroborated by findings from studies that assessed real product forms or placebo formulations of methods still in development (11). Thirdly, our education messages for injectable PrEP did not include the pharmacokinetic tail after cessation of use which leaves sub-optimal drug levels in the body hence increasing the risk of drug resistance if one acquired HIV and the likely need for a phase-out strategy to prevent this. We may therefore have overestimated preference for injectable PrEP. The mixed methods design we used is a strength as it enabled us to do more qualitative exploration of preferences among AGYW.

### Implications

Given the varied method preferences among AGYW, availability of recommended long-acting methods should be expedited to increase PrEP options and the likelihood of improved uptake of and adherence to preferred methods. However, drug resistance among individuals who stop using CAB-LA while still at increased risk of HIV acquisition, most likely true for a majority of PrEP users, will have to be monitored as data are still limited, and potential users must also be informed about this risk to make informed choices. The PrEP implant and HIV vaccine(s) would likely have the advantage of longer duration of protection when compared to CAB-LA, they will need to show similar or greater efficacy than CAB-LA, and more favorable attributes to warrant introduction. Any new MPTs that could protect against not only HIV, other STIs and/or unintended pregnancy, would be an attractive alternative method for AGYW.

Interventions to improve oral PrEP use, e.g., peer support, should be encouraged given that it is still the only widely available biomedical PrEP method in Uganda and several countries in Eastern and Southern Africa, a preferred option for a proportion of AGYW and more likely to be used by AGYW whose sexual partners are living with HIV or have unknown HIV status. After almost four decades of HIV prevention options being largely confined to the condom, health education messages about biomedical PrEP methods to individuals and communities will have to be re-designed for novel biomedical PrEP methods.

## Data availability statement

The original contributions presented in the study are included in the article/Supplementary material, further inquiries can be directed to the corresponding author.

## Ethics statement

The studies involving humans were approved by Uganda Virus Research Institute Research Ethics Committee and Uganda National council for Science and Technology. The studies were conducted in accordance with the local legislation and institutional requirements. Written informed consent for participation was not required from the participants or the participants’ legal guardians/next of kin because written informed consent was obtained from all participants including emancipated and mature minors aged 14–17 years who can consent for research participation as per national guidelines.

## Author contributions

YM: Conceptualization, Funding acquisition, Investigation, Methodology, Project administration, Visualization, Writing – original draft. IK: Data curation, Formal analysis, Investigation, Visualization, Writing – review & editing, Software. OK: Formal analysis, Investigation, Methodology, Software, Validation, Writing – review & editing. JL: Validation, Writing – review & editing, Data curation, Software. VM-K: Investigation, Resources, Writing – review & editing, Project administration, Validation. MP: Writing – review & editing, Investigation, Resources. KK: Methodology, Supervision, Writing – review & editing. AE: Methodology, Supervision, Writing – review & editing.
