# Corrigendum: Preference for novel biomedical HIV pre-exposure prophylaxis methods among adolescent girls and young women in Kampala, Uganda: a mixed methods study

**DOI:** 10.3389/fpubh.2025.1597921

**Published:** 2025-04-02

**Authors:** Yunia Mayanja, Ivy Kayesu, Onesmus Kamacooko, Jane Frances Lunkuse, Vincent Muturi-Kioi, Matt Price, Kyriaki Kosidou, Anna Mia Ekström

**Affiliations:** ^1^Medical Research Council/Uganda Virus Research Institute and London School of Hygiene and Tropical Medicine (MRC/UVRI & LSHTM) Uganda Research Unit, Entebbe, Uganda; ^2^Department of Global Public Health, Karolinska Institutet, Stockholm, Sweden; ^3^Child Health and Development Centre, School of Medicine, Makerere University, Kampala, Uganda; ^4^IAVI, Nairobi, Kenya; ^5^4IAVI, New York, NY, United States; ^6^Department of Epidemiology and Biostatistics, University of California, San Francisco, San Francisco, CA, United States; ^7^Centre for Epidemiology and Community Medicine, Region Stockholm, Stockholm, Sweden; ^8^Department of Infectious Diseases/Venhälsan, Södersjukhuset, Stockholm, Sweden; ^9^Department of Clinical Science and Education, Södersjukhuset, Stockholm, Sweden

**Keywords:** pre-exposure prophylaxis, biomedical HIV prevention, preference, adolescent girls and young women, Uganda, Eastern and Southern Africa

In the published article, there was an error in the Funding statement. The project code for the EDCTP funder was erroneously written as “3120” instead of “3102” and we listed project activities funded rather than use the generic funder statement.

The original text is, “The author(s) declare financial support was received for the research, authorship, and/or publication of this article. This work was funded by IAVI and made possible by the support of many donors including United States Agency for International Development (USAID). The full list of IAVI donors is available at http://www.iavi.org. IAVI also sponsored the study and therefore contributed to the study design, monitored the study and, representatives reviewed and approved all versions of the manuscript. The East Africa Consortium for Clinical Research/736 EACCR3 (CSA2020NoE-3120) under the European and Developing Countries Clinical Trials 737 Partnership (EDCTP2) provided funding for travel and supervision of this work as part of doctoral 738 studies at Karolinska institute”.

The correct Funding statement appears below.

