# Supplementary material for: Preference for novel biomedical HIV pre-exposure prophylaxis methods among adolescent girls and young women in Kampala, Uganda: a mixed methods study
Source: Front Public Health. 2024 May 23;12:1369256. doi: 10.3389/fpubh.2024.1369256 (PMC11153736; doi:10.3389/fpubh.2024.1369256)
Supplement: Supplementary file 1 [file Data_Sheet_1.PDF]

MRC Medical Research Council
Uganda Virus Research Institute
LONDON SCHOOL of HYGIENE &TROPICAL MEDICINE

EDUCATION MESSAGES FOR ENROLLED VOLUNTEERS

Study Title: *A prospective cohort study to assess knowledge and preferences for biomedical HIV prevention interventions and uptake of oral pre-exposure prophylaxis among adolescents and young Women at high risk of HIV infection in Kampala, Uganda. Version 2.0_30 May 2018*

We are going to give you information about available HIV prevention methods and those that we hope will be available for us to use in the future

1. Let us start with oral pre-exposure prophylaxis (PrEP) *(5 mins)*

- These are pills containing antiretroviral drugs. The government of Uganda plans to make it accessible to HIV negative people at risk of HIV infection.
- The tablets look like this (***Use a bottle of oral PrEP pills kept for education purposes and show tablets to volunteers***). Each bottle contains 30 tablets; it is enough to take you for one month.

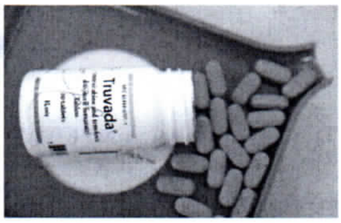

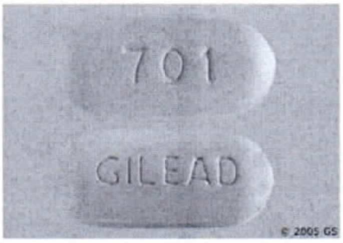

- Research has shown that if HIV negative people take these pills well, up to 90 out of every 100 people are protected from HIV.
- The dose is one pill taken once a day around the same time each day. The pills will only protect you if you adhere to the drugs very well.
- If you forget to take your pill and remember after 12 hours have passed, skip the pill for that day and take one the next day. Do not take two pills the next day.
- You cannot share pills with your peers because some tests need to be done to make sure you can take the pills. If you are already taking these pills, you still cannot share pills because each bottle contains enough pills for one person for the month.
- It is not advised to give your pills to HIV positive people or get pills from your HIV positive peers who are taking antiretroviral drugs (ART). The dose given to HIV negative people helps to protect against HIV infection while the dose for HIV positive people is to treat infection.
- Even though the pills reduce your chance of catching HIV, it is not 100% effective. You are therefore required to use condoms correctly and consistently at all times even when you have started taking the pills.

UGANDA VIRUS RESEARCH INSTITUTE ENTEBBE
RESEARCH ETHICS COMMITTEE
APPROVED
VALID UNTIL
20 JUN 2019
P. O. BOX 49, ENTEBBE
UGANDA

- All drugs have side effects. Some of the expected side effects of these pills include: nausea, vomiting, stomach pain, headache, weight loss, shortness of breath, muscle pain, getting tired too quickly but these usually go away within the first month of taking the drug. Some serious side effects such as reduced function of the kidneys may occur but these are very rare.
- You may be able to stop taking the pills if your risk for HIV infection reduces.
- It is best to use the pills in combination with other available HIV prevention methods such as condoms, regular HIV testing and counselling (HCT) and prompt treatment for sexually transmitted infections (STIs).
- The other benefit of these pills is that they are used as treatment for Hepatitis B infection.

In this study, you will be offered oral PrEP pills.

Question and Answer session (10 mins)

2. **Now let us talk about injectable antiretroviral drugs/ ARVs (Injectable PrEP); *5mins***

- Antiretroviral drugs can also be given as injections. Please note that injectable PrEP is still under research so is not yet available for use.
- The drug will be packed like this (***Demonstrate using a drug ampoule***) and will be given as an injection. It is slowly released into the body over a period of 2-3 months.

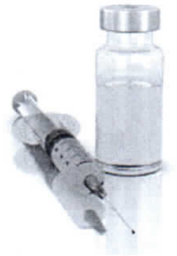

UGANDA VIRUS RESEARCH INSTITUTE ENTEBBE
RESEARCH ETHICS COMMITTEE
APPROVED
VALID UNTIL
20 JUN 2019
P. O. BOX 49, ENTEBBE
UGANDA

- Early research shows that injectable PrEP is safe and well tolerated by most people.
- The injection will be given once every 2-3 months to protect HIV negative people from HIV infection. You will need to keep your clinic appointments to receive injections as scheduled.
- The common side effects are: sleep problems, unusual dreams; mild nausea, vomiting, stomach pain, diarrhoea, liver problems, headache, dizziness, fever, fatigue, flu-like illness, mild skin rash, allergy, changes in the shape or location of body fat (especially in your arms, legs, face, neck, breasts, and waist).
- Injection site reactions include: pain, itching swelling and feeling hot were also common side effects noted in the trials. Rare side effects that have been noted include: severe flu-like illness, chills, pain, rash, depression and psychosis.
- You will still be required to use the available HIV prevention methods (condoms, HCT and prompt treatment of STIs.)

Please note that long acting injectable drugs are still in research and therefore not yet available for use in our community.

Question and answer session *(10 mins)*

3. **The third method we shall talk about is the vaginal microbicide ring (5 mins)**

- The vaginal microbicide ring also contains an antiretroviral drug called Dapivirine. Please note that the ring is also still under research so is not yet available for use.

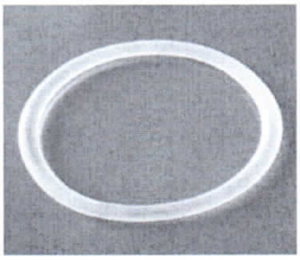

- The ring looks like this **(Demonstrate using a sample of the Dapivirine ring kept for education purposes)**. It is flexible and is inserted into the vagina.
- When inserted, it slowly releases the drug into the body over a period of one month.
- Research shows that if HIV negative people adhere well to the ring, up to 56% are protected from HIV.
- The ring is changed once every month; you are able to insert and remove it by yourself so you can get a 3 months' supply to reduce the number of times you go to a clinic. Once you insert it well, you will not feel it.
- Some of the expected side effects while using the ring include: inflammation, irregular bleeding between menstruation periods, reduced frequency of menstruation (more than 35 days before the next period), reddening, or swelling of the opening of the uterus (cervix), urinary tract infection, vulvovaginal candidiasis, itching of vulval skin, loss of bladder control, headache; pain during sex, and pelvic pain.
- You are still encouraged to use other available HIV prevention methods such as condoms, HCT and PEP even when using the ring.
- The ring does not protect you from pregnancy or sexually transmitted infections

Please note that the vaginal ring is still in research and therefore not yet available for use in our community.

Question and answer session *(10mins)*

4. **<u>We shall now talk about the implant</u>**

UGANDA VIRUS RESEARCH INSTITUTE ENTEBBE
RESEARCH ETHICS COMMITTEE
APPROVED
VALID UNTIL
20 JUN 2019
P. O. BOX 49, ENTEBBE
UGANDA

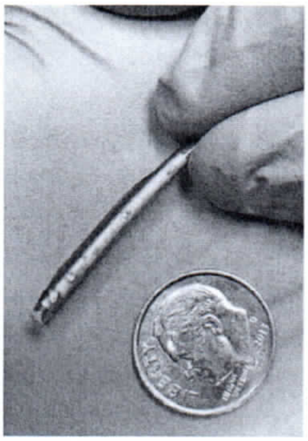
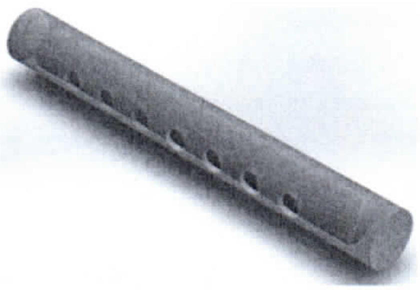

- The antiretroviral implant would look like this. **(Demonstrate using the pictures above and the implanon contraceptive rod).** It is a small rod inserted under the skin of the arm, the same way as the contraceptive implant.
- An implant is easily inserted and removed. Once well inserted, you will go about your activities normally.
- When inserted, it slowly releases the drug into the body over a period of several months.
- You will need to visit a clinic once in several months to a year to have the implant changed
- Some of the expected side effects while using the implant will be reactions such as pain and swelling at the site of insertion.
- This product is still being developed for early research and we hope that more information about it will be available in future.
- The antiretroviral implant will not protect you from pregnancy or sexually transmitted infections

Please note that the implant is still in research and therefore not yet available for use in our community.

Question and answer session *(10mins)*

5. <u>The last method we shall talk about is the HIV vaccine *(5 mins)*</u>

- An HIV vaccine will work like any of the common vaccines we already have such as hepatitis B, Measles, Tetanus vaccines etc. It would prepare your body's defence mechanism to fight HIV infection in case you were exposed to HIV.

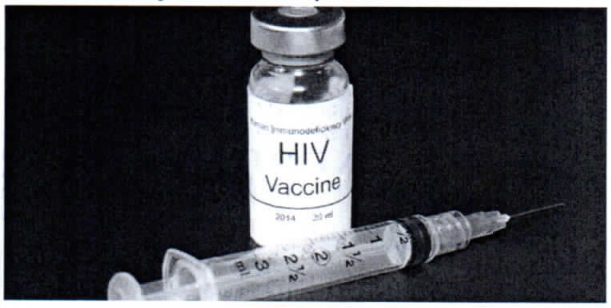

UGANDA VIRUS RESEARCH INSTITUTE ENTEBBE
RESEARCH ETHICS COMMITTEE
APPROVED
VALID UNTIL
20 JUN 2019
P. O. BOX 49, ENTEBBE
UGANDA

- Please note that an HIV vaccine is not yet available for use. It will be packaged in a vaccine vial like the other vaccines we already have such as Hepatitis B vaccine, DPT and Yellow Fever vaccines. (***Demonstrate using an empty Hepatitis B vaccine vial)***.
- You will receive the vaccine through an injection given into the muscle.
- The vaccines will be given as 4 doses: a start dose, at 1 month, 3 months and 6 months. Researchers are still studying HIV vaccines; they will inform us if this kind of vaccination schedule works and if these will be the only doses you will receive to be protected for life.
- Expected side effects will be reactions at the injection site such as pain, swelling, itching, soreness. More research will show what other effects will be.
- It is expected that the HIV vaccine will offer protection against HIV infection. It will however not protect you from pregnancy or other sexually transmitted infections.
- You will need to get tested regularly for HIV at the site where you got the vaccine and not at other HIV testing facilities.

Please note that HIV vaccines are still in research and therefore not yet available for use in our community.

Question and answer session *(10mins)*

UGANDA VIRUS RESEARCH INSTITUTE ENTEBBE
RESEARCH ETHICS COMMITTEE
APPROVED
VALID UNTIL
20 JUN 2019
P. O. BOX 49, ENTEBBE
UGANDA
