# Supplementary material for: Preference for novel biomedical HIV pre-exposure prophylaxis methods among adolescent girls and young women in Kampala, Uganda: a mixed methods study
Source: Front Public Health. 2024 May 23;12:1369256. doi: 10.3389/fpubh.2024.1369256 (PMC11153736; doi:10.3389/fpubh.2024.1369256)
Supplement: Supplementary file 2 [file Data_Sheet_2.PDF]

IPAD study: Assessment of Understanding of Education Messages- EM-AoU Version1.0

SCR ID: SCR|__|__|__|**S [SCRID]**   Study IPD|__|__|__|**[IPID]**   Visit No: |__|__|. |__| **[VNO]**

Visit Date: |___|___| /|___|___|___| /|___|___|___|___| **[VDATE]**

Interviewer instructions: *Ask the volunteer the following questions. If the volunteer thinks the statement is true cross T, if false cross F.*

**1. The pills that prevent HIV infection will only protect you if you adhere to them very well.** **[OPREP]**	□ T □ F
**2. The injectable drug that prevents HIV infection is slowly released into the body over a period of 2-3 months.** **[INJPREP]**	□ T □ F
**3. The vaginal ring when inserted into the vagina, slowly releases the drug into the vagina over a period of one month.** **[VRING]**	□ T □ F
**4. An HIV vaccine would be given once every year for the period when you are at risk of HIV infection** **[HVAC]**	□ T □ F
**5. An implant that prevents HIV infection is still under research and therefore not yet available for use in our community** **[FUVISIT]**	□ T □ F

Score : ___/ 5   **[SCORE]**

List questions failed: _______________**[QNFAIL1]** *(Do not fill if score = **5 / 5**)*

Provide additional information ***for all questions answered incorrectly****. Ensure that the volunteer demonstrates understanding of failed questions*

Administer the Preference CRf at visits 2 and 4

Form completed by: ___________ [INITIALC]   Date: |___|___| /|___|___|___| /|___|___|___|___|

Form Reviewed by: ____________ [INITIALR]   Date: |___|___| /|___|___|___| /|___|___|___|___|

1st Data Entry: ________________ [INITIALE1]   Date: |___|___| /|___|___|___| /|___|___|___|___|

2nd Data Entry: _______________ [INITIALE2]   Date: |___|___| /|___|___|___| /|___|___|___|___|
