# Supplementary material for: Preference for novel biomedical HIV pre-exposure prophylaxis methods among adolescent girls and young women in Kampala, Uganda: a mixed methods study
Source: Front Public Health. 2024 May 23;12:1369256. doi: 10.3389/fpubh.2024.1369256 (PMC11153736; doi:10.3389/fpubh.2024.1369256)
Supplement: Supplementary file 3 [file Data_Sheet_3.PDF]

Supplementary File 3: Assessment of Understanding of Education Messages on five Biomedical HIV Prevention Methods

Questions Asked (N=264) *	Response	Scored (n)	Scored (%)
The pills that prevent HIV will only protect you if you adhere to them well.	True	264	100
The injectable drug that prevents HIV is slowly released into the body.	True	264	100
The vaginal ring when inserted into the vagina slowly releases the drug into the vagina.	True	261	98
An HIV vaccine would be given once every year for the period when you are at risk.	False	224	85
An implant that prevents HIV infection is still under research and therefore not available.	True	261	98

* Assessment records for one participant were missing.
